# Critical factors influencing visitor emotions: analysis of “restorativeness” in urban park visits in Fuzhou, China

**DOI:** 10.3389/fpubh.2023.1286518

**Published:** 2023-11-21

**Authors:** Yu Wu, Jian Liu, Jay Mar D. Quevedo, Huishan Cheng, Kunyong Yu, Ryo Kohsaka

**Affiliations:** ^1^College of Landscape Architecture and Art, Fujian Agriculture and Forestry University, Fuzhou, China; ^2^Graduate School of Agricultural and Life Sciences, The University of Tokyo, Tokyo, Japan; ^3^College of Forestry, Fujian Agriculture and Forestry University, Fuzhou, China

**Keywords:** urban green space, emotional health, environmental quality, preference, restorative environment

## Abstract

**Objective:**

To date, a comprehensive analysis of urban green space (UGS) visitors’ emotional remains largely unexplored. In this study, we focus on how UGS environmental preferences, restorativeness, other physical factors (sound, air, and thermal environments), and individual characteristics affecting visitor emotions. Such a comprehensive analysis would allow relevant practitioners to check the environmental quality of UGSs and improve certain conditions to promote visitor emotions.

**Methods:**

A total of 904 questionnaire responses with concurrently monitored physical factors were analyzed by independent sample *t*-tests, one-way ANOVA and path analysis.

**Results:**

The thermal evaluation had the largest impact on positive emotions (β = 0.474), followed by perceived restorativeness (β = 0.297), which had β values of −0.120 and −0.158, respectively, on negative emotions. Air evaluation was more effective for increasing positive emotions (β = 0.293) than reducing negative emotions (β = −0.115). Sound evaluation also had similar results (β = 0.330 vs. β = −0.080). Environmental preference significantly influenced only positive emotions (β = 0.181) but could still indirectly impact negative emotions. Moreover, objective physical factors can indirectly affect visitors’ emotions by enhancing their evaluations..

**Conclusion:**

The influence of different UGS environmental factors on visitors’ emotions vary, as does their impacts on positive versus negative emotions. Positive emotions were generally more affected than negative emotions by UGS. Visitor emotions were mainly influenced by physical and psychological factors. Corresponding suggestions are proposed for UGS design and management in this study.

## 1 Introduction

### 1.1 Background

The current speed, and magnitude of global urbanization are unprecedented. By 2050, more than two-thirds of the global population is projected to reside in cities (1). This issue is particularly important in China (2). At the city scale, urban heat is a prominent environmental concern that impacts most cities in China (3) and affects daily human activities and health (3). Other environmental problems, such as air pollution and noise also threaten the health of urban residents (4). Although the home quarantine policy during the COVID-19 pandemic has alleviated air and noise pollution, this reversed after lockdown was lifted (5, 6). Furthermore, overcrowded housing and work pressures threaten residents’ mental health (7), which has worsened during the COVID-19 pandemic (8) and may pose a substantial public health risk (9). These pieces of evidence have indicated that urban residents are facing a series of health threats.

In recent decades, growing evidence has shown that urban green space (UGS) is associated with a range of important benefits to human health (10, 11). For example, exposure to UGS can have positive effects on individuals, including reducing anxiety and stress, improving emotions and attention, and positively influencing behavior (12–14). Additionally, the presence of UGS has been linked to lower incidences of disorders, as well as physical and psychological benefits during heat stress episodes (15). UGS can also mitigate environmental problems associated with resident health, including air pollution and noise (16). These previous findings are evidence that UGSs have become indispensable for promoting city resident health.

Previous studies have focused primarily on the impacts of UGS on personal disease, physical activity, social interactions, and psychological stress. However, a comprehensive analysis of visitor perspectives and the crucial UGS factors that affect visitor emotions has been rarely performed. Emotional health can reduce the risk of depression and anxiety, improve interpersonal relationships, enhance self-awareness and self-control, and improve work efficiency (17, 18). It implies that emotional health is essential to an individual’s health and overall quality of life. UGS is also considered one of the important places for regulating individual emotions. Therefore, it is more important to understand what has happened in UGS and how UGS affects the emotions of visitors. This information can provide detailed references for UGS designers or managers to improve the quality of UGS and improve visitor emotions.

Additionally, there is sporadic evidence directly or indirectly indicating the impact of UGS characteristics on emotional health, yet it remains necessary to provide comprehensive references for relevant practitioners (such as urban planners, park designers, park managers, etc.) to balance and coordinate different UGS environmental characteristics to improve visitor emotions. We may know that certain UGS characteristics can affect individual emotions, but it is still unclear which type of characteristics are primary, and which are secondary. In addition, some studies have used remote sensing, social network, or simulation experiments to obtain research data, which can to some extent replace field data, but cannot ensure that the collected data fully matches the actual situation (19–21). For example, UGS environments simulated through photos, videos, or virtual reality typically do not include other physical factors that match them (such as climate, air quality, sound, etc.). The experimental participants are not guaranteed to be the actual users of UGS either. It is for this reason why certain UGS studies still use field research methods. Some studies have also explored the impact of the environment on individual behavior and health through real-time measurement of objective environmental parameters combined with questionnaire surveys (22). These parameters can provide objective references for UGS managers to take corresponding measures in a timely manner. Despite practical and theoretical urgency, these studies rarely focus on visitor emotional health.

Therefore, based on existing research, we propose relevant hypotheses on the key factors affecting the emotions of UGS visitors. A combination of questionnaire surveys and real-time monitoring of physical factors were conducted to collect data. Afterwards, the proposed hypotheses were validated using methods such as path analysis. We discuss the relationships between various environmental factors and reveal the β values of these factors on emotions, providing a comprehensive perspective for relevant practitioners to balance and coordinate various UGS environmental factors through UGS design and management.

### 1.2 Conceptual framework

Emotion is defined as a short-term state that is directly related to environmental stimuli, which can be accompanied by a series of physiological reactions and behaviors (23). Ulrich et al. (24) proposed that the primary response of humans to environmental stimuli is emotions. Although moods and emotions are frequently used interchangeably, the constructs they represent are closely related but distinct phenomena. Most theorists have agreed that moods endure longer than emotions and proposed that emotions are usually displayed or expressed behaviorally whereas moods are not (25). Therefore, the visitor emotions in this study aims to explore is a matter of short-term perception and more associated with human real-time sensations of multiple environmental stimuli on UGS environmental levels.

**Figure 1:**
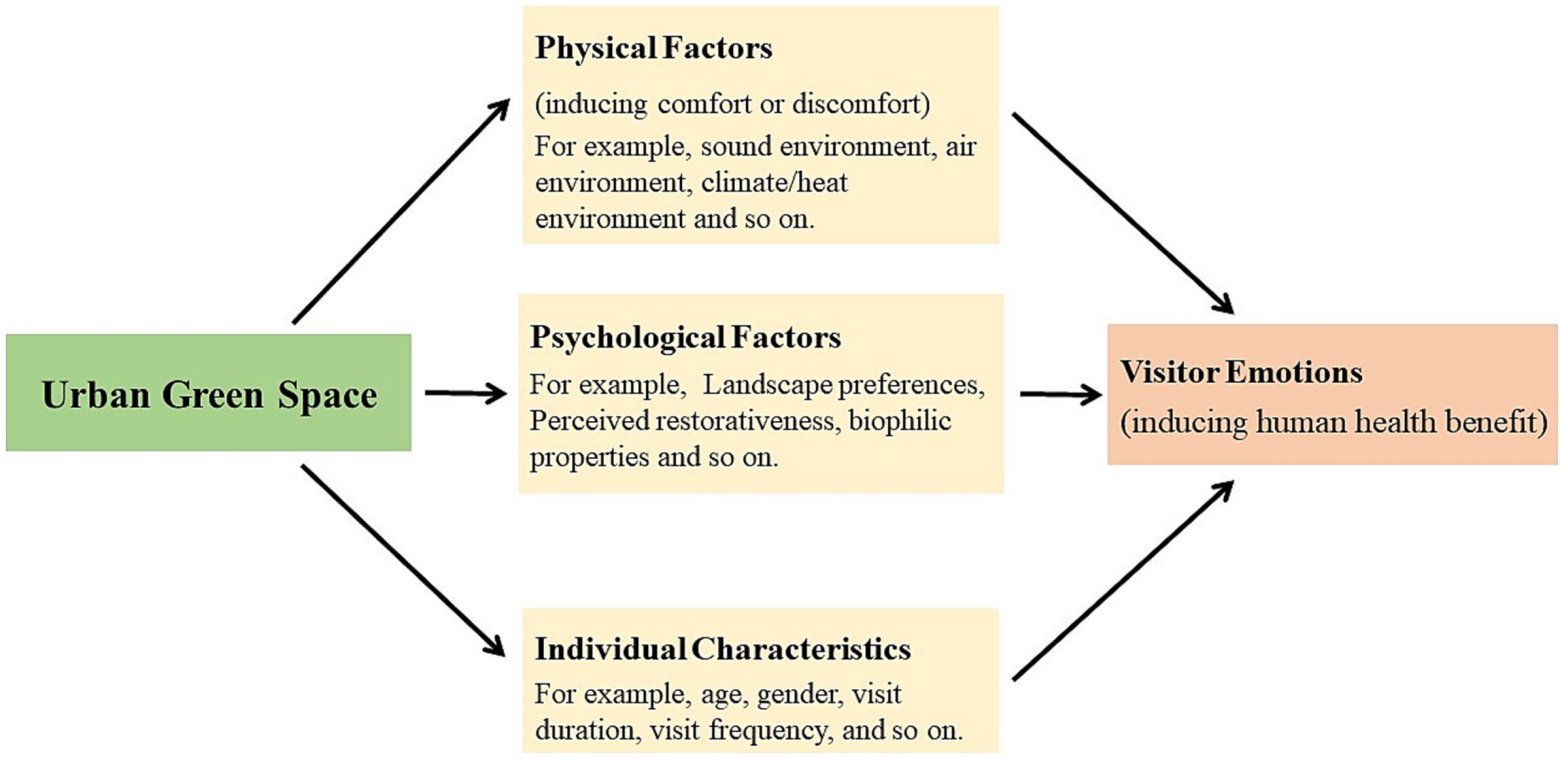
Three domains of pathways linking greenspace to visitors emotions.

The emotions of UGS visitors involved various studies in many fields. Broadly speaking, there are following 3 main streams of studies on visitor emotions. One stream of studies primarily focused on the exploration of emotions based on environmental psychological theory, such as restorative environment, landscape preference theory, and etc. Another stream of visitor emotions studies focused on physical environmental factors that induce comfort or discomfort and affect their emotions during visiting. Using thermal environment as an example, a majority of them suggests that physical thermal environment can cause thermal discomfort for tourists and affect their emotions in hot summer. In addition, some studies reported the impact of individual characteristics (like gender, age, visit frequency and etc.) on visitor emotions. Therefore, we reviewed relevant research in these 3 fields and summarized the critical factors that affect visitor emotions (Figure 1).

#### 1.2.1 Psychological factors

In UGS, visitor emotions are often closely associated with the “restorative environment” theory in environmental psychology. The term “restorative” refers to the processes of renewing or recovering resources or capacities that have become diminished or depleted while meeting the demands of everyday life (26). The environment with this “restoration” is called a restorative environment (27). Natural environments are usually considered typical restorative environments. In crowded cities, limited UGS has become the valuable restorative environment. Hartig et al. (28) summarized four qualities characterize of restorative environmental experience: being away, fascination, extent, and compatibility.

Stress reduction theory (SRT) and attention restoration theory (ART) are two of the most famous restoration theories. According to SRT (24), interactions with nature lead to stress recovery, resulting in reduced physiological arousal and negative affect, as well as increased positive affect. In contrast, ART emphasizes the restoration of one’s ability to concentrate or to direct attention (29). ART contends that the capacity to focus or to direct attention requires cognitive effort; thus, it is prone to fatigue. The restoration of directed attention can occur when involuntary attention is engaged, which is considered effortless and does not require cognitive effort.

The integration of SRT and ART has also been proposed (29). Directed attention fatigue can lead to negative emotional consequences, similar to stress (30). Jung et al. (31) found that improved attention is not driven by emotion. Instead, attention recovery resulting from reduced attention fatigue through restorative environmental intervention may improve mood, as negative emotions (e.g., irritability and anxiety) may be symptoms of attention fatigue. Previous research supports the integration of attention and emotion theories (32, 33). Marselle et al. (34) observed a positive effect of perceived restorativeness on emotional health. However, Sato and Conner (35) did not find that fascination with restorative quality was related to negative emotions. Similarly, Hung & Chang (36) only found a significant impact of landscape preference on positive emotions. In general, these findings support an association between perceived restorativeness and the positive aspects of emotional health; however, the association with negative affect requires further investigation. UGS is the primary restorative environment in city areas; therefore, it is compelling to suggest that the restorativeness of UGS will affect visitors’ emotions, as described in SRT.

Most previous studies have also suggested that environmental health benefits may involve individual preferences (37, 38). Pazhouhanfar and Kamal (39) reported that four predictors of visual landscape preferences (coherence, complexity, legibility, and mystery) help enhance restorativeness in UGS. Subsequently, Liu et al. (40, 41) also demonstrated that the positive effects of landscape preference on restorative evaluation and health benefits in urban parks, but did not include legibility. These health benefit assessments overlap with emotional reactions (e.g., restoring vitality, calming, and concentration). Furthermore, recent research has revealed a correlation between individual preferences for flower colors and positive emotional responses, such as feelings of enhancement and relaxation (42). As UGSs are the primary location in which visitors can interact with plants and flowers within city limits, it is likely that similar patterns exist in UGS. Based on this, we recognize the close correlation between landscape preference and restorative environments, and further speculate that landscape preference may be a crucial component of the mechanism by which UGS influences a visitor’s emotional well-being.

Some biodiversity studies also share the similar viewpoint. Biodiversity can be considered a measure of an environment’s complexity (belong to landscape preference) (43) and has been found to be associated with positive emotional well-being, and greater perceived restorativeness (34, 44, 45). The biophilia hypothesis also supports similar views. They believe that humans are naturally prefer nature and subconsciously want to be close to the natural environment (46, 47). A more natural environment is more favored and aesthetically valued by humans, and is beneficial for human health and well-being (48). The biophilic properties of the environment were closely related to individual landscape preferences and emotions in UGS (36). However, Marselle et al. (34) and Nghiem et al. (49) found that it is not the perception of the environment’s naturalness/nature relatedness that leads to greater emotional well-being, but that perceived naturalness/nature relatedness offers opportunities for a restorative experience which then contributes to positive emotional well-being. Therefore, although the biological properties/naturalness of the environment have been found to be related to emotional health, existing research results tend to suggest that perceived restorativeness and landscape preference have a more direct impact on individual emotions.

#### 1.2.2 Physical factors

Plants improve the physical environment (including reducing carbon emissions, naturally cooling and purifying the air) through photosynthesis, transpiration and purification and promote human health and comfort (50). As the area with the largest proportion of plants in the city, UGS plays an important role in improving urban heat islands, noise problems, and air pollutions (16, 51, 52). At present, a majority of studies targeted at mitigating thermal discomfort under hot weather conditions as it would induce discomfort and affect visitor emotions and health. For example, Hami et al. (53) summarized the effects of plant characteristics, planting methods, and arrangement methods on individual improving thermal comfort. Salata et al. (54) proposed that implement more vegetation and built roofs and roads using cooling materials. Other studies have explored the correlation between noise/soundscape, air quality and human health in UGS (4, 42, 55, 56). Only few studies have identified the importance of these physical environment factors in the emotional health of UGS visitors. For example, Park et al. (57) confirmed the correlation between thermal comfort and positive emotions in urban and forest environments. Zhang et al. (58) confirmed that thermal sensation, restorative perception and landscape features could significantly affect individuals’ emotions in summer. Yu et al. (59) found that environmental noise had a significant harmful health impact, involving emotions such as annoyance, arousal, and pleasantness, whereas Zhou et al. (60) also demonstrated the negative impact of noise on citizens’ emotions in a study of UGS soundscapes. By investigating the psychological status of residential green space users during the COVID-19 pandemic, Li et al. (61) found that perceived air pollution acted as a mediating variable in the relationship between residential green spaces and symptoms of anxiety and depression. At present, there is still a lack of direct evidence to demonstrate the impact of the physical factors provided by UGS on visitor emotions. But it is clear that visitors’ subjective evaluation of these physical factors can affect their emotions.

Certain studies have collected objective environmental measurements based on data collected near their respective study locations (42). The parameters from the nearest meteorological station and environmental monitoring point to the UGSs to some extent reflect physical factors. However, different landscape structures within the parks not only create different microclimate environments (58), but also affect the diffusion of car exhaust, thereby affecting air quality (4). The distance between the research area and the measurement station may also cause deviation. These indirect measurements may not fully represent the actual physical factors of the UGS where people stay. To accurately reflect the actual environment experienced by visitors to a UGS, it seems better to use mobile equipments to do the real-time measurements combined with questionnaire surveys.

#### 1.2.3 Individual characteristics

Visitor emotions are personal in green space experiences. It may due to differences of the perception and use in UGS by different genders or ages of tourists (62). Mouly et al. (63) found that women and older people may gain slightly greater healthy benefits from greenspace. This result was consistent with other studies (48, 62, 64, 65). However, Fu et al. (66) suggested that courtyard space landscape has boosted slightly better in men emotions than in women, and people over the age of 30 experienced better mood benefits. The reason for these inconsistent results may be that these studies are based on different national backgrounds and use different age groups, survey methods and different research materials. In addition, the duration and frequency of visit also be related to visitor emotions. A study on the use of UGS among the old people found that the more frequent and longer the exposure to UGS, the better the physical and mental health of tourists (67). Similarly, the results of two other studies also support the positive correlation between visit duration and tourists’ mental health benefits (49, 68, 69). At the same time, higher frequency and longer duration of UGS visits are related to tourists’ higher familiarity and place attachment (70). Liu et al. (41) have also found a positive impact of place attachment on the health benefits of green spaces. Therefore, these results supported the view that the more frequently and more longer time tourists are exposed to UGS, the more beneficial it is to their emotions. Furthermore, other personal characteristics have been found to affect the health benefits that tourists receive in UGS, such as their health status, physical activity intensity, behavioral types, and even the childhood experiences (71–73).

### 1.3 Hypothetical structure

This study aims to identify critical factors that affect the emotions of UGS visitors by empirical analysis. It discusses the interrelationships of these factors, and compares their impact on visitor emotions to propose new data regarding the impacts of various UGS features on visitors’ emotional health, offering valuable insights for formulating strategies to improve the comprehensive quality of UGS.

**Figure 2:**
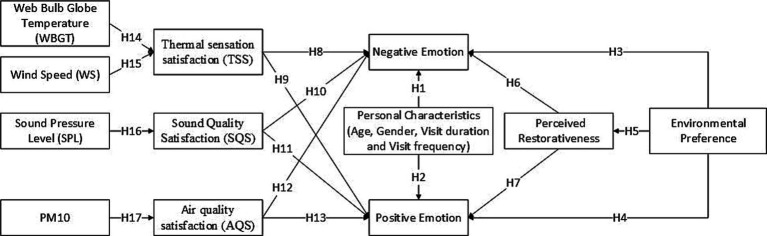
Proposed hypothetical framework for the path model used in this study.

Firstly, visitor emotions have been divided into positive and negative aspects to determine the key factors associated with these two aspects of emotions. Subsequently, according to our conceptual framework proposed in section 1.2, using a created hypothetical structure (Figure 2) to discuss critical factors that influence visitor emotions. These critical factors include psychological factors, physical factors, and individual characteristics aspects. The specific content is as follows:

We assumed that the visitors negative/positive emotions are significantly influenced by individual characteristics (H1/H2), phycological factors (environmental preference H3/H4, and perceived restorativeness H6/H7) and physical factors (subjective evaluation of thermal sensation H8/9, sound quality satisfaction H10/11, and air quality H12/13), respectively. Among them, environmental preference indirectly affects visitor emotions by directly influencing perceived restorativeness (H5). Thermal sensation satisfaction (TSS) is directly affected by wet bulb globe temperature (WBGT; H14) and wind speed (WS; H15). Sound pressure level (SPL) and Air quality satisfaction (AQS) are directly affected by sound pressure level (SPL; H12) and PM10 concentration (H13), respectively. Table 1 provides a detailed description of the corresponding assumptions and references. The selection basis and acquisition method of various indicators are explained in detail in sections 2.2, 2.3, and 2.4.

**Table 1:** Major hypotheses in the proposed conceptual framework.

Category	Hypothesis	Description	References
Individual characteristics	H1/H2	Personal characteristics influence visitor negative/positive emotions	
	H1A/H2A	Age influence visitor negative/positive emotions	(65, 66)
	H1B/H2B	Gender influence visitor negative/positive emotions	(48, 66)
	H1C/H2C	Frequency of visit influences visitor negative/positive emotions	(67)
	H1D/H2D	Duration of visit influences visitor negative/positive emotions	(69)
Psychological factors	H3/H4	Environmental preference influences visitor negative/positive emotions	(36)
	H5	Environmental preference affects visitor emotions by directly influencing perceived restorativeness	(39, 41, 74)
	H6/H7	Perceived restorativeness influences visitor negative/positive emotions	(31, 34)
Physical factors	H8/H9	Evaluation of thermal environment influences visitor negative/positive emotions	(42, 75)
	H10/H11	Evaluation of sound quality influences visitor negative/positive emotions	(4, 59, 60)
	H12/H13	Evaluation of air quality influences visitor negative/positive emotions	(4, 76)
	H14	Wet bulb globe temperature (WBGT) influences evaluation of thermal environment	(77, 78)
	H15	Wind speed (WS) influences evaluation of thermal environment	(79, 80)
	H16	PM_10_ concentration influences evaluation of air quality	(81, 82)
	H17	Sound pressure level (SPL) influences evaluation of sound environment	(60, 83)

## 2 Methodology

### 2.1 Study area

**Figure 3:**
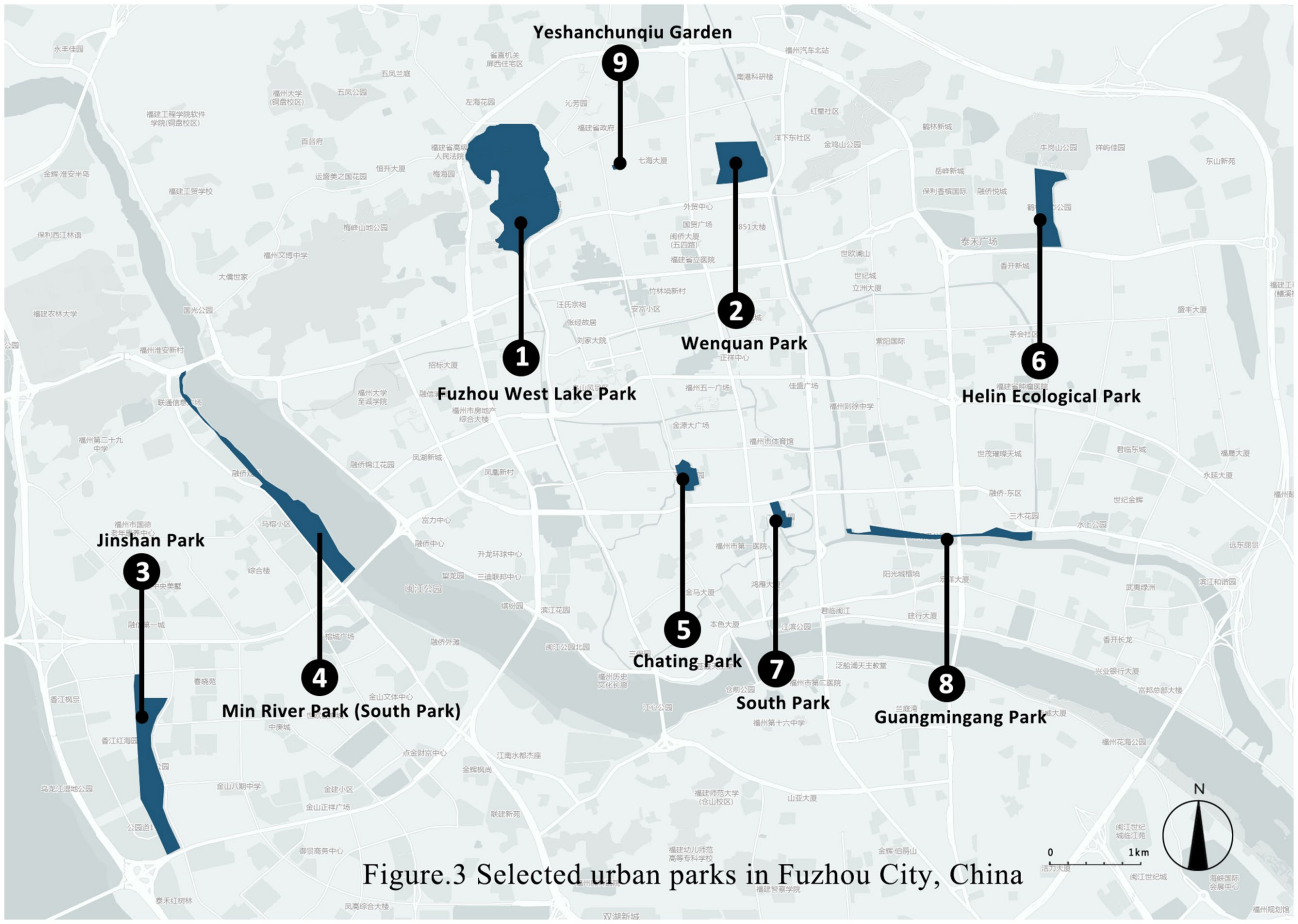
Selected urban parks in Fuzhou city, China.

This study was conducted in Fuzhou, China. As of 2021, the total area of parks and green spaces in Fuzhou City reached 14,199.95 ha, with an urban park green space area of 5,426.12 ha that included 166 urban parks (Fuzhou Ecological Environment (84)). The *per capita* green space area of parks in Fuzhou is 14.82 m^2^, approximately the same as the Chinese average (14.87 m^2^). Figure 3 shows the locations of the 9 surveyed parks, and their characteristics are summarized in Table 2. These parks are located in the urban area, but their locations, shapes and surrounding environments varies. The area ranges from 3.43 to 42.51 ha, and the percentage of green and water area also varies. It leads to the diversity of landscape characteristics, physical environment and visitor characteristics in different parks. In addition, although it includes newly built and well-established parks, their maintenance is well.

**Table 2:** Information on survey parks.

Name	Built year	Area (ha)	Plant (%)	Water (%)	Landscape characteristics	Location	Surrounding environment	Maintenance status
Fuzhou West Lake Park	1954	42.51	41	50	Have a large lake and various aquatic/terrestrial plants and animals	City center	Adjacent to arterial road, surrounded by residential and commercial areas	Well maintained
Wenquan Park	1997	13.17	65	11	A European style park with various terrestrial plants and birds	City center	Adjacent to sub-arterial road, surrounded by bustling commercial district	Well, 2 sites are under repair
Jinshan Park	2004	31.16	69	21	A belt park with a river and various aquatic/terrestrial plants and animals	Main urban area edge	Adjacent to sub-arterial road, surrounded by residential areas	Well, 3 sites are under repair
Min River Park	2002	27.4	62	2	A belt park on the edge of the Min River; with wide views and various aquatic/terrestrial plants and animals	Main urban area	Adjacent to arterial road and Min river, surrounded by residential areas	Well maintained
Chating Park	1996	7.02	64	18	Chinese classical garden with a small lake and various plants and birds	Main urban area	Adjacent to sub-arterial road, surrounded by residential and commercial areas	Well maintained
Helin Ecological Park	2020	16.67	64	10	An ecological park with a river and various plants	Main urban area edge	Adjacent to arterial road, surrounded by new residential areas	Well maintained
South Park	2016	3.09	53	17	Chinese classical garden with some green terrestrial plants and birds	Main urban area	Adjacent to sub-arterial road, surrounded by residential areas	Well maintained
Guangmingang Park	1998	14	64	6	A belt park on the edge of inland river; wide views and various aquatic/terrestrial plants and birds	Main urban area edge	Adjacent to arterial road and inland river, surrounded by residential areas	Well maintained, 2 sites are under repair
Yeshanchunqiu Garden	2020	3.43	68	3	Chinese classical garden with some green terrestrial plants	City center	Adjacent to sub-arterial road, surrounded by residential and commercial areas	Well maintained

Made based on (85) and on-site survey situation.

Among them, Fuzhou West Lake Park, a classical garden located in the city center, is the largest and has a large lake accounting for half of the area. It is rich in animal and plant resources, and the landscape here are diverse. The surrounding area is bustling commercial and residential areas, with a large flow of vehicles and people. Wenquan Park is also located in the city center, is less than one-third the size of Fuzhou West Lake Park and features a European-style design as well as hot spring characteristics. It has 11% of the water area and varies animals and plants. Although the surrounding sub-arterial roads have relatively low traffic flow, they are located in bustling commercial areas with high pedestrian flow. Yeshanchunqiu Park is also located in the city center and the smallest park, belonging to street-level green spaces. It is mostly green trees and grasslands, with no lakes or rivers. In contrast, Jinshan, Guangminggang and Helin Ecological Park are located at the boundary of the central urban area. The surrounding areas are mostly residential areas. Both Jinshan Park and Helin Ecological Park have a river. But their landscape styles are vastly different. There are many Chinese classical garden style facilities such as pavilions, corridors, and arch bridges in Jinshan Park, while the structures in Helin Park are mostly in modern style. Due to the early construction of Jinshan Park, the plants there are denser and the animal resources are more abundant. Guangminggang Park is adjacent to the inland river, with a wide view and various aquatic/terrestrial plants and birds. Min River, South, and Chating Park are located between the city center and the city edge with different sizes and landscape styles. Min River Park is adjacent to the largest river in the city (Min River). The park has a wide view and various aquatic/terrestrial plants and animals. Although Chating Park only covers one fourth of the area of Min River Park, it has been built for a long time and is rich in animal and plant resources. South Park has the smallest area and contains some bird and plant resources. A diverse set of parks was selected to ensure data collection from various attributes of UGS environments.

### 2.2 Research design

This study used a combination of real-time mobile measurement and questionnaires to collect data. The surveys were conducted in July and August 2022, from 7 AM to 6 PM on days with sunny weather. The surveys of Yeshanchunqiu Garden, Wenquan Park, Chating Park, and South Park were conducted simultaneously (two parks were surveyed at the same time in 1 day), while the surveys of the other 5 parks were conducted separately (one park was surveyed in 1 day). The survey of 9 parks ended in one round of survey (7 days), and a total of 3 rounds of survey were conducted (21 days). Six staff members were divided into several investigation groups, each being equipped with the required measuring instruments and assigned a responsible area. These areas cover the entire park and there are no duplicate areas. The group members walked along park roads and asked visitors about their willingness randomly. After obtaining the permission, they distributed questionnaires to the visitors and used mobile instruments to measure and record real-time physical environment data. A total of three rounds of surveys was conducted in a day, at 7 AM, 11 AM, and 3 PM. Each round of survey takes approximately 2 to 3 h to complete.

**Figure 4:**
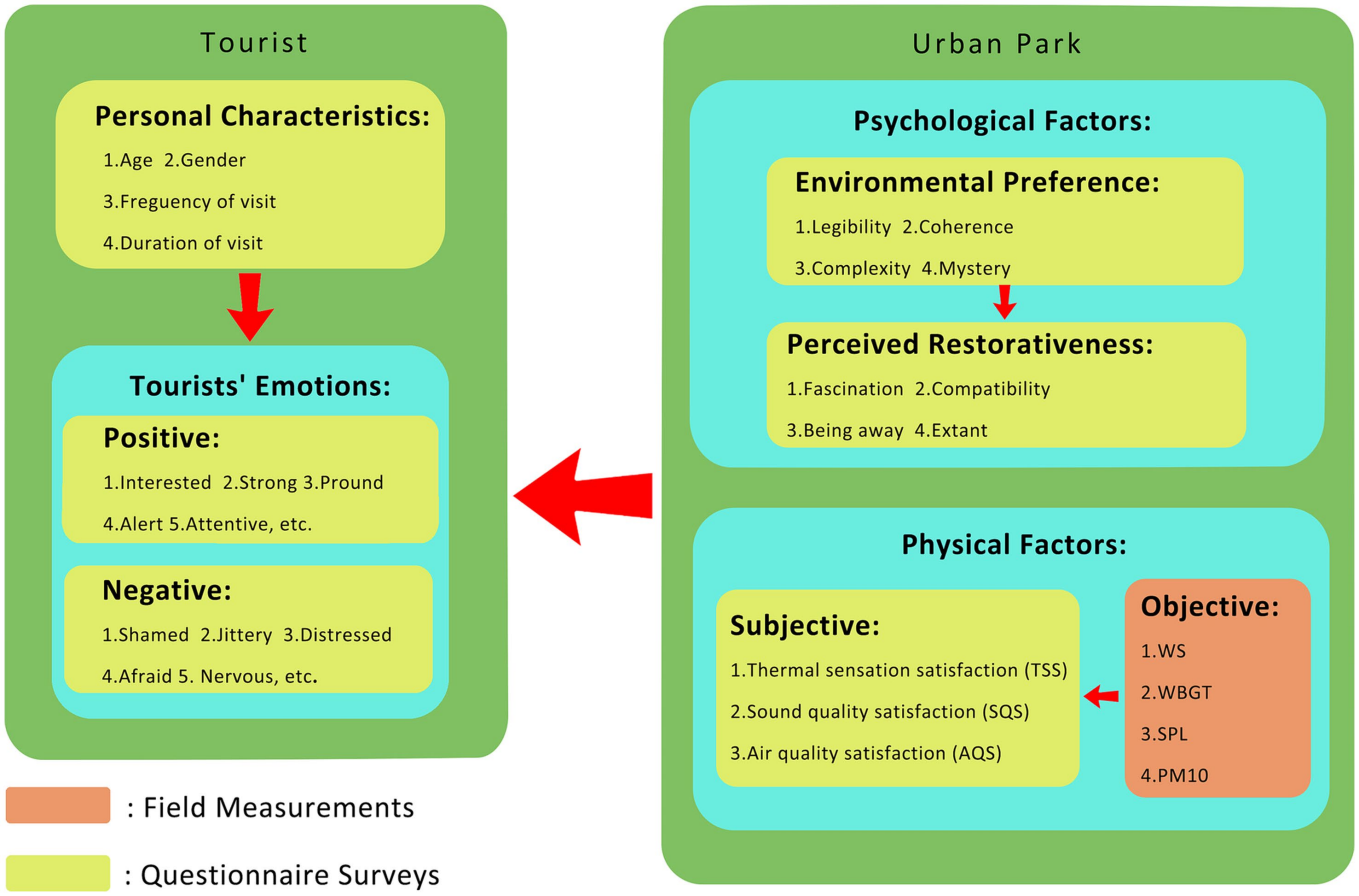
Outline of the methods used to collect different types of data.

Face-to-face questionnaires were used to gather data from respondents regarding their personal characteristics, emotions, environmental preferences, perceived restorativeness, and satisfaction with physical factors (sound, air, and thermal environments). Movable instruments were used simultaneously during the interviews to measure and record the air, and thermal environmental parameters near the respondents. The sound parameters were measured after the questionnaires to avoid the noise generated during the interviews affecting the results. Figure 4 shows the methods used to collect the various types of data. After all data collection was completed, IBM SPSS Statistics ver. 23 was then used to analyze and determine the impact of personal characteristics on visitor emotions. Path analysis was used to explore the relationships and path coefficients of environmental preferences, perceived restorativeness, and objectively and subjectively measured physical factors on visitor emotions.

### 2.3 Field measurements

This study used mobile instruments to measure objective physical factors such as WBGT, WS, SPL, and PM10. The data obtained were matched with the time and location that the respondents answered questionnaire. To ensure the physical environment conditions when and where they were located was accurately reflected. These indicators were selected for the following reason:

The objective thermal environment indicators included the WBGT and WS. WBGT and physiological equivalent temperature (PET) are widely adopted thermal indices and have been used in discussions of outdoor thermal environments (77, 78). The WBGT index was calculated using the formula: WBGT = (0.7 × Tw) + (0.2 × Tg) + (0.1 × Ta), where Tw, Tg, and Ta are the wet-bulb, black globe, and dry-bulb temperatures, respectively. Compared with the WBGT, PET indicators include some non-meteorological factors, such as amount of clothing and activity, which cannot be directly measured by instruments and may not be helpful for reviewing the objective UGS environment. However, WBGT does not consider wind factors. To comprehensively measure the objective thermal environment, we added WS as an indicator (79, 80). The A-weighted SPL is currently the most widely used sound measurement index as its characteristic curve is close to the auditory sensation characteristics of the human ear. This measurement is closely related to individual evaluations of the sound environment used in previous studies (60). Thus, A-weighted SPL was used as an objective acoustic environment indicator in this study. The concentration of airborne particulate matter (PM) is usually used as a measure of air quality, including PM2.5 and PM10 concentrations (81, 82). PM2.5 refers the concentration of particles with a diameter of less than 2.5 μm in the ambient air. Similarly, PM10 refers to the concentration of particles with a diameter of less than 10 μm in the ambient air. By comparing their concepts, it was found that PM10 already includes PM2.5. To avoid data redundancy, PM2.5 did not included in this study. In addition, PM2.5 particles are particularly small and difficult for humans to perceive, whereas PM10 particles are more easily sensed (81). PM10 is better suited for predicting visitors’ air quality satisfaction and emotions.

Table 3 lists all the measurement instruments and their specifications, which was assembled to measure outdoor WBGT, WS, PM10, and SPL. Almost all of the data were recorded continuously at the pedestrian level throughout the survey period. After stabilizing, all measuring instruments began recording. WS, WBGT, and SPL recorded the average data values for 5 min, while PM10 recorded the average values of two measurements to minimize measurement errors. The SPL data were collected after conducting the face-to-face survey to avoid interference from the conversation.

**Table 3:** Specification details of the measurement instruments.

Instrument	Measurement parameter	Measurement range	Accuracy
Kestrel Hand-held weather station NK5500	Wind speed	0.6–40 m/s	±3%
Taiwan Hengxin thermal index meter AZ8778	WBGT	0 ~ 50°C	±3.5°C
CEM air quality detector DT96	PM_10_ concentration	0 ~ 2000ug/m^3^	±5%
MASTECH multi-function environment detector MS6300	Sound pressure level(A)	0 ~ 130 dB(A)	±1.5 dB

### 2.4 Questionnaires

The interviewers introduced themselves as students from a university and asked the visitors whether they wanted to be interviewed (They can withdraw at any time). Many visitors expressed willingness; however, some people declined to participate. The visitors who agreed to be interviewed were informed of the study’s anonymity and purpose, and how the data would be processed and used. To ensure that they understood this agreement, participants were given a consent form to read and sign. In most cases, the respondents filled out the questionnaires with interviewer assistance only if questions arose. A few older respondents asked the interviewers to read the questions and record their responses. The following sections are the components of the questionnaire.

#### 2.4.1 Perceived restorativeness

“Restorativeness” is commonly used to describe the degree of restoration or restorative quality of an environment (86). In this study, we used the perceived restorativeness scale (PRS), proposed by Hartig et al. (28), which effectively measures the psychological recovery effect of the environment on individuals. According to the ART, four co-acting qualities characterize the experience of a restorative environment: being away, fascination, extent, and compatibility (28).

In this study, PRS was assessed using a four-item scale, which has been used in former studies with UGS and identified to have good reliability and validity (87, 38). The questions raised were as follows: “Spending time here gives me a good break from my daily work (Being away),” “The environment here has sufficient content and structure that it can occupy my mind for a long period (Extent),” “I think the environment here is very charming and attracted me (Fascination),” and “I would like to stay here longer, as I can enjoy myself in this scene (Compatibility).” This test scale was measured using a 5-point Likert scale ranging from 1 (fully disagree) to 5 (fully agree).

#### 2.4.2 Environmental preference

The landscape preference matrix (88) proposes information gain as a determinant of landscape preference. The first domain of information acquisition and ease of understanding yields two attributes: coherence (ease of understanding of immediate surroundings) and legibility (ease of orientation for movement). The second domain, the potential for exploration, also yields two attributes: complexity (number of different elements in immediate surroundings) and mystery (promise of more information while venturing further). Therefore, we used four indicators (coherence, legibility, complexity, and mystery) to measure environmental preferences. Combined with the Kaplan’s definitions of various dimensions of preferences (88) and refer to Chinese scales (41, 89), it consists of four questions: (1) Coherence: What do you think of the coherence of this place (intensity of coherence and organization of the landscape environment in terms of lines, textures, shapes, colors, or materials)? (2) Legibility: What do you think of the degree of landscape harmony and unity here? (3) Complexity: What do you think of the landscape richness and diversity here? (4) Mystery: To what extent does the landscape here make you feel mysterious and want to explore further? Respondents were required to provide a score that ranges from 1 (very slight or not at all) to 5 (extremely high) for each question.

#### 2.4.3 Emotion

Watson et al. (90) developed the two-factor positive and negative affect schedule (PANAS) model to measure emotional states in different time frames (e.g., moment, today, past, year, and general). Ten adjectives representing positive affect (PA) and 10 adjectives representing negative affect (NA) were included in this study. The PANAS scale is one of the most widely used emotion scales available (58, 91) and has good reliability and validity and is suitable for the Chinese population (92). Thus, we used 10 adjectives for PA: interested, strong, proud, attentive, alert, enthusiastic, inspired, determined, active, and excited. The 10 adjectives for NA were shame, hostile, distress, nervous, fear, jittery, afraid, guilt, irritability, and upset. A 5-point Likert scale was used to measure the score for each emotion, ranging from one (very slight or not at all) to five (extremely high).

#### 2.4.4 Personal characteristics and physical environmental evaluations

This section recorded the respondents’ characteristics, including gender, age, and duration as well as frequency of visits to the surveyed environment. Based on the age classification methods of two studies on green space emotions in China (65, 66) and WHO (93), we divided the respondents into three age groups: (1) young people (25 years old or younger), (2) middle-aged people (26–50 years old), and (3) older adults (51 years of age and older). The physical factors evaluations were also evaluated using a 5-point Likert scale, ranging from one (Very dissatisfied) to five (Very satisfied), including thermal sensation satisfaction (TSS), sound quality satisfaction (SQS), and air quality satisfaction (AQS).

### 2.5 Data analysis

All data were coded in Microsoft Excel and analyzed using IBM SPSS Statistics ver. 23 and Mplus ver. 8.3. IBM SPSS Statistics ver. 23 was used to perform statistical analyses, including descriptive analysis, t-tests, and one-way analysis of variance (ANOVA), using the collected questionnaire responses. Path analysis is an extension of multiple regression models and can be considered a special case of structural equation modeling (94). Path analysis has been widely applied in many fields, including biology, psychology, and sociology (22, 95). Path analysis was applied using Mplus to determine the effects of environmental preferences, perceived restorativeness, and objectively/subjectively measures physical factors on visitor emotions. We set the number of bootstrap samples to 1,000, with a 95% confidence level.

## 3 Results

### 3.1 Descriptive statistics

We obtained results from 904 respondents of all age groups, including 555 males and 349 females (Table 4). Males slightly outnumbered females. Most of the respondents reported spending less than 30 min or over an hour in the park, with a visitation frequency of fewer than five times per week. Only 19.4% of respondents visited the park more than five times a week, 22.3% of whom would remain in the park for over 1 h.

**Table 4:** Personal characteristics of the respondents.

Variable	Category	Number	Percentage
Gender	Male	555	61.4
	Female	349	38.6
Age	≤25	422	46.7
	26–50	297	32.9
	≥51	185	20.5
Duration of visit	<15 min	366	40.5
	15–30 min	175	19.4
	31–45 min	53	5.9
	46–60 min	108	11.9
	>60 min	202	22.3
Frequency of visit in a week	≤1	462	51.1
	2–4	267	29.5
	≥5	175	19.4

According to the results of independent sample t-tests and one-way ANOVA (Table 5), we found significant differences in visitors’ positive emotions based on age (*F* = 3.474, *p* < 0.05), visit duration (*F* = 6.395, *p* < 0.001), and frequency of visits (*F* = 17.73, *p* < 0.001). Only visitors’ ages had a significant impact on their negative emotions (*F* = 3.519, *p* < 0.05). However, neither the positive nor negative emotions of the respondents were significantly influenced by their gender.

**Table 5:** Differences in respondents’ emotions by respondents’ characteristics.

	Variable	Positive emotions		T/F	P	Negative emotions		T/F	P
		Mean	SD			Mean	SD		
Gender	Male	4.018	1.07	−0.07	0.940	3.096	1.094	−1.1	0.273
	Female	4.023	1.114			3.169	0.775		
Age	≤25	3.986c	0.053	3.474	0.031	3.035b	0.058	3.519	0.030
	26–50	3.953c	0.069			3.227a	0.044		
	≥51	4.205ab	0.064			3.162	0.054		
Duration of visit	<15	3.863bcd	1.137	6.395	0.000	3.128	1.142	0.07	0.991
	15–30	4.198ae	0.985			3.099	0.952		
	31–45	4.440ae	0.688			3.149	0.658		
	46–60	4.215ae	0.914			3.104	0.672		
	>60	3.936bcd	1.184			3.144	0.914		
Frequency of visit in a week	≤1	3.814bc	1.195	17.73	0.000	3.138	0.724	0.043	0.957
	2–4	4.217a	0.918			3.126	0.801		
	≥5	4.263a	0.904			3.124	0.983		

Letters “a” to “g” indicate a significant difference by the LSD post-test results. For example, in the age group, “a” represents a significant difference between the current group and the first group (≤18).

Table 6 summarizes the objective/subjective physical factors in the surveyed parks, including WBGT, WS, PM10, SPL, TSS, SQS, and AQS. The respondents were most satisfied with the air quality, followed by sound quality. Thermal sensation was rated as the worst. By combining these results with the objective data, we can better understand the outcomes. The average PM10 value was 14.16, which is significantly lower than China’s first-class national standard (50 mg/m3) and explains why the respondents were highly satisfied with the air quality. The average SPL value was 57.7, which is slightly lower than the 60 dB standard for Class 2 urban land according to Chinese urban environmental noise standards. The average values of WBGT and WS were 29.99°C (WBGT >29°C indicates a heat stress environment) and 0.98 m/s, respectively. The heat-stressed environment and weak winds likely contributed to the respondents giving the worst evaluation of their satisfaction with thermal sensation.

**Table 6:** Measured physical factors (*N* = 904).

Measured physical factors	Mean	SD	Range
Objective:			
WS(m/s)	0.98	0.98	0.0–7.0
WBGT(°C)	29.99	1.45	26.7–37.1
SPL(dB A)	57.70	7.41	42.0–85.1
PM_10_(mg/m^3^)	14.16	7.08	2–38
Subjective:			
TSS	3.26	1.10	1–5
SQS	3.38	0.77	1–5
AQS	3.69	0.70	1–5

### 3.2 Path analysis

**Figure 5:**
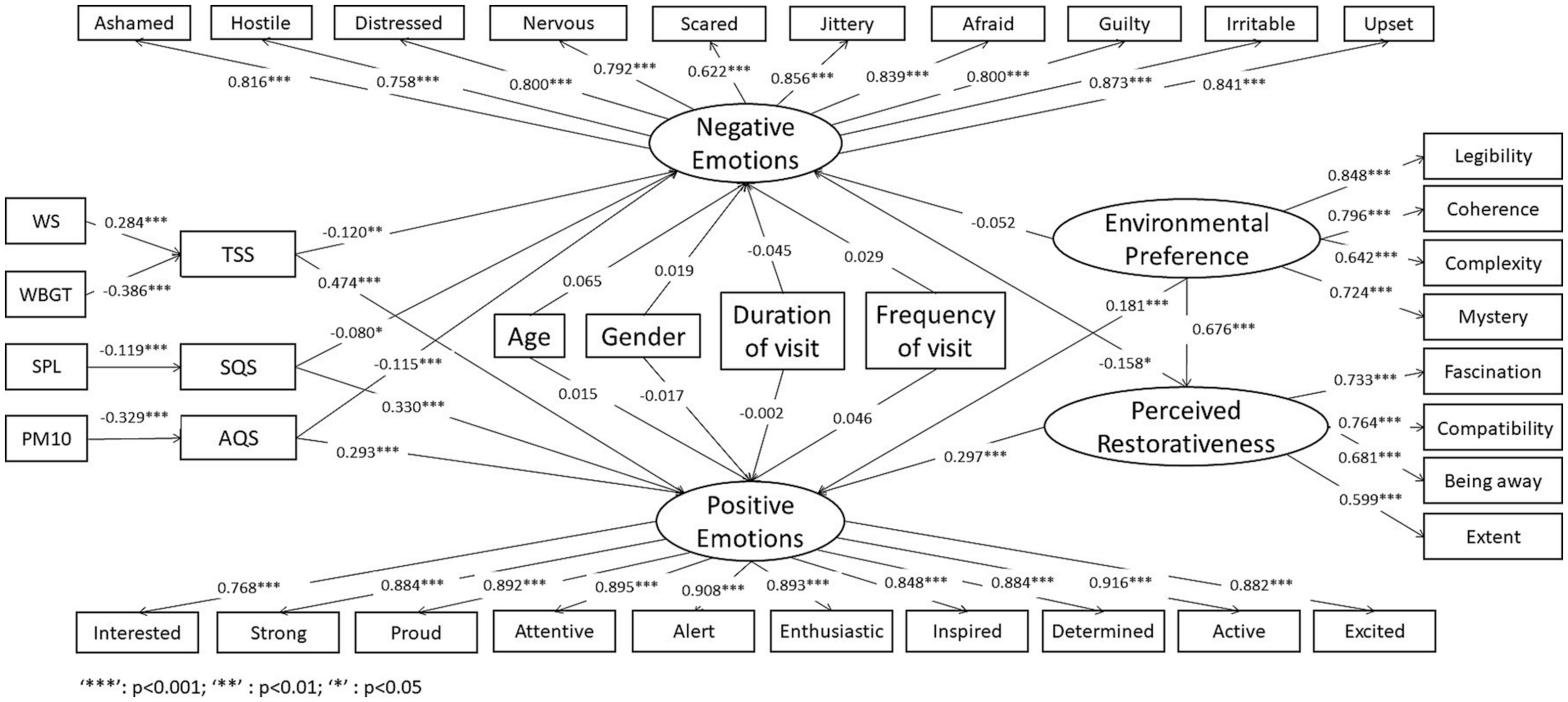
Estimated coefficients values for individual factors in the formulated path model.

Figure 5 shows the final path model, including the estimated correlation values for the individual factors. The standardized coefficients (β) were used in the model to facilitate comparisons of the effect degree of each path. High values indicate a strong causal relationship between the dependent and independent variables, whereas low coefficient values indicate a weak relationship. Positive coefficients indicate that the value of the independent variable increased with an increasing dependent variable, whereas negative coefficients imply that the independent variable decreased with an increasing dependent variable. Symbols represent the significance levels of the independent variable’s impact on the dependent variable (*: *p* < 0.05; **: *p* < 0.01; ***: *p* < 0.001). The path model constructed herein illustrates the relationships among visitor emotions, personal characteristics environmental preferences, perceived restorativeness, and physical factors.

Cronbach’s alpha for the PANAS (PA) scale was 0.980 (Table 7), which is greater than 0.7. The corrected item-total correlation (CITC) of 0.980 was greater than 0.5, and the Cronbach’s alpha for all deleted items were less than the dimension reliability, indicating good reliability for this dimension. Similarly, the reliability tests for the PANAS (NA; Cronbach’s alpha = 0.948), environmental preference (Cronbach’s alpha = 0.832), and perceived restorativeness (Cronbach’s alpha = 0.784) scales were also successful.

**Table 7:** Reliability analysis of the survey questionnaires (*N* = 904).

Measurement scales	Items	Mean	SD	CITC	Cronbach’s alpha if item deleted	Cronbach’s alpha
PANAS (PA)-positive emotions	Interested	3.867	1.172	0.815	0.980	0.980
	Strong	4.090	1.137	0.911	0.977	
	Proud	4.012	1.191	0.912	0.977	
	Attentive	4.053	1.144	0.915	0.977	
	Alert	4.089	1.184	0.925	0.977	
	Enthusiastic	4.021	1.196	0.912	0.977	
	Inspired	3.990	1.196	0.878	0.978	
	Determined	4.149	1.119	0.909	0.977	
	Active	4.018	1.204	0.929	0.977	
	Excited	3.910	1.267	0.899	0.978	
PANAS (NA)-negative emotions	Ashamed	2.690	1.114	0.791	0.943	0.948
	Hostile	3.241	1.238	0.753	0.944	
	Distressed	3.178	1.229	0.784	0.943	
	Nervous	2.930	1.196	0.775	0.943	
	Scared	3.568	1.267	0.627	0.950	
	Jittery	3.097	1.177	0.830	0.941	
	Afraid	3.371	1.195	0.824	0.941	
	Guilty	3.427	1.181	0.793	0.942	
	Irritable	2.738	1.126	0.843	0.940	
	Upset	3.001	1.169	0.816	0.941	
Perceived restorativeness	Fascination	3.623	0.771	0.650	0.700	0.784
	Compatibility	3.472	0.750	0.643	0.705	
	Being away	3.474	0.798	0.564	0.745	
	Extent	3.336	0.796	0.511	0.772	
Environmental preference	Legibility	3.705	0.808	0.747	0.749	0.832
	Coherence	3.559	0.817	0.716	0.763	
	Complexity	3.261	0.938	0.581	0.831	
	Mystery	3.491	0.763	0.619	0.806	

Table 7 indicated all the average of 10 positive emotions were higher than that of negative emotions. Among positive emotions, determined (4.149) and strong (4.090) had higher mean scores. As for negative emotions, ashamed (2.690), irritable (2.738), and nervous (2.930) emotions had lower mean scores. The average scores of every item of perceived restorativeness and environmental preference were high, ranging from 3.261 to 3.705. It is consistent with the current research view that UGS has high restorativeness and environmental preference attributes, and is beneficial to increasing positive emotions and reducing negative emotions.

Table 8 presents a comparison of the goodness-of-fit indices for the model with generally accepted model fit criteria. The formulated model is a reasonably good representation of the relationships, as its values meet the requirements for *X*^2^/df, comparative fit index (CFI), Tucker-Lewis index (TLI), and root mean square error of approximation (RMSEA), which are commonly used to evaluate the goodness-of-fit of path models.

**Table 8:** Acceptance criteria and calculated values of various goodness-of-fit indices for the model.

Goodness-of-fit index	*X*^2^/df	CFI	TLI	RMSEA
Acceptance value	2.0–5.0	>0.90	>0.90	≤0.08
Model value	4.500	0.913	0.906	0.062

The respondents’ positive/negative emotions were influenced not only by their environmental preference (EP) and perceived restorativeness (PR), but also by physical factors, including TSS, SQS, and AQS (Table 9). Personal characteristics (gender, age, duration of visit and frequency of visit), as covariates, have no significant effect on visitor emotions. In terms of influencing positive emotions, the β values of PR and EP were 0.297 and 0.181 (*p* < 0.001), respectively; while those of TSS, SQS, and AQS were 0.474, 0.330, and 0.293 (*p* < 0.001), respectively. These results indicate that the TSS, SQS, and PR had more effect on visitor positive emotions than EP and AQS. The partial mediating effect of PR between EP and positive emotions was demonstrated by a total effect of 0.381, including an indirect effect of 0.201 and a direct effect of 0.181 (95% confidence interval that excludes 0; Table 10).

**Table 9:** Results of the path analysis.

Dependent variable	Independent variable	Estimate	S.E.	Est./S.E.	*p*	β
TSS	WS	0.316	0.040	7.955	0.000	0.284
	WBGT	−0.292	0.025	−11.747	0.000	−0.386
SQS	SPL	−0.012	0.004	−3.254	0.001	−0.119
AQS	PM_10_	−0.032	0.003	−9.808	0.000	−0.329
PR	EP	0.557	0.040	14.001	0.000	0.676
Positive emotions	Gender	−0.027	0.038	−0.709	0.478	−0.017
	Age	0.015	0.031	0.502	0.616	0.015
	Duration of visit	−0.001	0.015	−0.069	0.945	−0.002
	Frequency of visit	0.047	0.031	1.514	0.130	0.046
	PR	0.417	0.065	6.385	0.000	0.297
	EP	0.209	0.052	4.044	0.000	0.181
	TSS	0.342	0.019	17.626	0.000	0.474
	SQS	0.341	0.030	11.340	0.000	0.330
	AQS	0.334	0.034	9.885	0.000	0.293
Negative emotions	Gender	0.035	0.056	0.625	0.532	0.019
	Age	0.075	0.046	1.606	0.108	0.065
	Duration of visit	−0.025	0.021	−1.154	0.249	−0.045
	Frequency of visit	0.033	0.046	0.713	0.476	0.029
	PR	−0.251	0.103	−2.436	0.015	−0.158
	EP	−0.068	0.085	−0.801	0.423	−0.052
	TSS	−0.098	0.030	−3.207	0.001	−0.120
	SQS	−0.093	0.046	−2.012	0.044	−0.080
	AQS	−0.148	0.048	−3.123	0.002	−0.115

**Table 10:** Results of the mediation analysis.

	Effect	Estimate	S.E.	Est./S.E.	P	β	Bootstrapping 95% CI	
							Lower	upper
EP-PR-positive emotions	Total	0.441	0.045	9.867	0.000	0.381	0.368	0.517
	Indirect	0.232	0.038	6.115	0.000	0.201	0.177	0.303
	Direct	0.209	0.052	4.044	0.000	0.181	0.123	0.295
EP-PR-negative emotions	Total	−0.208	0.055	−3.812	0.000	−0.159	−0.301	−0.119
	Indirect	−0.14	0.06	−2.343	0.019	−0.107	−0.243	−0.046
	Direct	−0.068	0.085	−0.801	0.423	−0.052	−0.203	0.074

EP did not have a significant impact on negative emotions. Accordingly, EP can still have an indirect effect on negative emotions by influencing PR, with a total effect of −0.159 and an indirect effect of −0.107 (Table 10). Significant negative correlations were found between negative emotions and PR (β = −0.158, *p* < 0.05), TSS (β = −0.120, *p* < 0.01), AQS (β = −0.115, *p* < 0.01) and SQS (β = −0.080, *p* < 0.05). PR, TSS, and AQS had more effect on negative emotions, compared to SQS.

Overall, compared to reducing negative emotions, urban park environments are more influential for enhancing visitors’ positive emotions. The satisfactions of physical factors (TSS, SQS, and AQS) played important roles in visitors’ positive emotions. For example, the impacts of TSS, SQS and AQS (0.474, 0.330 and 0.293, respectively) on positive emotions were higher than those of PR and EP (0.297 and 0.181, respectively). In addition, despite PR having the largest impact on negative emotions, the influences of TSS, SQS, and AQS cannot be disregarded (−0.158 vs. −0.120, −0.080, and −0.115, respectively). The results of the path analysis (Table 9) indicate that the objective physical environment indicators were significantly correlated with the corresponding subjective physical environmental satisfaction. For example, WS had a significant positive effect on TSS (β = 0.284, *p* < 0.001), whereas WBGT had a significant negative effect on TSS (β = −0.386, *p* < 0.001). Similarly, SPL had a significant negative effect on SQS (β = −0.119, *p* < 0.01), and PM10 had a significant negative effect on AQS (β = −0.329, *p* < 0.001).

## 4 Discussion

### 4.1 Summary of main findings

In this study we successfully formulated a path model that provides an integrated view of how various factors affect visitor emotions in UGS. These factors include individual characteristics, psychological factors (perceived restorativeness, and environmental preferences), and physical factors (noise, air quality, thermal environment). The path analysis also successfully determined the relationships among the relevant subjectively and objectively measured physical factors in UGSs. Compared to the hypotheses H1-H17 proposed in Section 1.3, all hypotheses are valid except H1, H2, and H3. In other words, visitor emotions were mainly influenced by physical and psychological factors, and the impact of personal attributes did not significant. Notably, this study provides a comprehensive understanding of the contributions of all factors to visitor emotions. The influence degree of different UGS environmental factors on visitors’ emotions varies, as does their impact degree on positive versus negative emotions. Visitors’ positive emotions were generally more affected by UGS than negative emotions. Specifically, PR and TSS have stable and relatively substantial effects on both positive and negative emotions. AQS and SQS are more effective at increasing positive emotions than at reducing negative emotions. Moreover, EP was found to significantly influence positive emotions only, but can indirectly impact negative emotions to some extent, albeit to a lesser degree. In addition, objective physical factors such as WBGT, WS, SQL, and PM10 can affect visitor emotions by increasing their satisfaction with the corresponding physical factors. Based on the results of all critical factors affecting the visitors’ positive/negative emotions, we provide various suggestions for designers and managers. The information provides valuable insights for UGS planners to develop effective strategies for enhancing human emotional health in UGSs.

### 4.2 The effect of critical environmental factors on UGS visitor positive emotions

The results indicate that TSS, SQS, AQS, EP, and PR have a significant positive impact on visitor positive emotions. Among them, the β value of TSS on visitor emotions was the largest, followed by SQS and PR. Unsurprisingly, TSS has a significant and maximum positive impact on tourists’ positive emotions. This may be due to the hot summer background highlighting the impact of tourists’ hot feelings on positive emotions. Hot environments may inhibit visitors’ positive emotions, such as excitement, activity, and attention. Based on the negative impact of WBGT on TSS and the positive impact of WS on TSS, we suggest reducing WBGT and increasing WS to improve UGS. As UGS designers, increasing greenery and water areas, and setting up sunshade facilities such as pavilions and trees are effective ways to reducing WBGT (53, 96). The larger the crown diameter, the higher the tree, the richer the tree diversity can achieve better cooling effect (97–99). Planting with appropriate distances between the trees and using a combination of trees and grass can serve as a solution for cooling UGS (100, 101). Using the surfaces and materials that have a higher albedo is also a method of reducing the environmental heat (102).

The surprising finding in this study is that SQS had a slightly larger effect on positive emotions than PR (0.330 vs. 0.297). Previous studies on the influence of the sound environment on PR, EP, human comfort, and behaviors (83, 103, 104) only indirectly indicated the necessity of maintaining a good sound environment in parks for visitors’ emotional health, which is achievable by reducing noise. However, the findings provide us with a new perspective that maintaining a satisfactory sound environment could be more important than creating a restorative environment. Therefore, we suggest that UGS managers be more concerned about the noise issue in UGS. In addition, designers should also be concerned about setting up corresponding soundproof green belts with large and high tree crowns near some noise sources, such as urban main roads (105).

PR is known to have positive impacts on physical and psychological health (51, 106, 107). Creating a being away, fascinating, extensive, and compatible restorative environment is conducive to improving personal mental health (34, 108). The fascination, compatibility and being away of UGS gradually attract tourists, make them getting away from the daily life and feel active, attentive and determined. In the study, we obtained a similar result: improving the PR helped mobilize individuals’ positive emotions. It can be achieved by increasing the degree of greenery, biodiversity, and nature relatedness/connectedness towards nature (49, 109). Previous studies have suggested that watching colorful plants can make people feel uplifted and relaxed, especially orange, yellow, red, white, and blue (42). The colorful plants may attract involuntary attention from visitors, leading to the occurrence of directed attention restoration (i.e., enhanced PR) and thus obtaining emotional benefits. From the perspective of biodiversity, people generally have poor biodiversity-identification skills (110), and colorful plants may help visitors perceive biodiversity to some extent, thereby contributing to their emotional health (45). Moreover (111), found that blue space is more effective in improving the feeling of being away and increasing positive emotions, and proposed suggestions for combining blue and green space. Blue green spaces provide more natural resources than single blue or green spaces, exhibiting higher biodiversity and enhancing visitors’ PR and positive emotions (44, 49, 112). The calm lake combined with green tree shading creates a peaceful and harmonious beautiful environment, attracting visitors to the landscape and feeling comfortable, breaking away from their daily hustle and bustle. Therefore, we suggest that designers keep the high levels of greenery, biodiversity, and naturalness, and consider appropriately combining water bodies and colorful plants in UGS.

EP and AQS only had small effects on visitors’ positive emotions. The results confirmed that AQS had a positive impact on positive emotions, whereas PM10 had a negative effect on air quality satisfaction. These findings are consistent with the effects of air quality indicators (e.g., PM10 and PM2.5) on human health, as proposed by Shi et al. (20) and Wang et al. (97). Although we did not include PM2.5 in this study, it cannot be disregarded (113), as it exhibits a common trend with PM10. Designers can plant tree species with stronger dust reduction effects near urban roads, such as cypress, elm, papyrus, and masson pine. This method can isolate some noise as well as block airborne particles. Moreover, we found that EP had the smallest effect on positive emotions, but indirectly impacted positive emotions by influencing individuals’ restorative perceptions. Therefore, the impact of EP cannot be underestimated. These findings are consistent with those of previous studies, indicating that readable, coherent, complex, and mysterious environments were advantageous for human emotional health (39, 41). Among them, biodiversity can be regarded as a measure of environmental complexity (43). Based on our research findings, it can be concluded that biodiversity can benefit visitor emotions through TSS, PR, and EP. Therefore, improving the biodiversity of UGS is a good method. Water bodies are also often considered beneficial for landscape preferences (114), which is consistent with our proposed proposal to construct UGS based on water bodies.

### 4.3 The effect of critical environmental factors on UGS visitor negative emotions

TSS, SQS, AQS, and PR have a significant negative impact on visitor negative emotions; however, the results of β are differed from those of positive emotions. We could only find that PR, AQS, and TSS had significant effects on negative emotions that decreased in that order. Not surprisingly, PR and TSS influenced negative emotions, respectively. Urban heat island and busy daily life caused urban residents’ weakness, irritability and upset emotions. But as they enter a UGS where the urban thermal environment improved, it may make them feel satisfied and improve their emotions. The fascination of UGS attracts tourists, allowing them to escape the pressure of daily life and feel relaxed and at ease, in order to reduce their negative emotions such as nervous, irritability, and upset. These findings of Marselle et al. (115), Salata et al. (54), Zhang et al. (58), and Korpela et al. (107) directly and indirectly support our viewpoints. Designers and managers still need to prioritize measures to improve PR and TSS quality of UGS: such as improving greenery, nature relatedness/connectedness towards nature, and tree diversity. These have been proven to help improve PR and alleviate the heat island effect, thereby reducing negative emotions among visitors (49, 97, 109).

However, the β value of AQS surprisingly ranked second. We speculate that this may be because visitors are more sensitive to the health threats posed by air pollution. Health threats can lead to negative emotions such as fear and anxiety. UGS designers and managers can take measures to improve air environment of UGS, which are potentially required to reducing negative emotions among visitors. Although satisfaction with the sound environment had a significant negative impact on negative emotions, the influence of the sound environment was weak, and far weaker than its influence on positive emotions. The reason for this phenomenon remains unclear. However, we conclude that a good sound environment is still conducive to reducing negative emotions among visitors. EP did not have a direct significant impact on negative emotions but could indirectly affect negative emotions through restorative perception.

The results suggest that park environments may influence individuals’ positive emotions more than their negative emotions. This finding is similar to that of Sato and Conner (35) who showed that fascination with the restorative environment was positively correlated with larger positive effects and was unrelated to negative impacts. Hung & Chang (36) also only found a significant impact of landscape preference on positive emotions and did not find a significant impact on negative emotions. Nevertheless, despite their limited impact, the various environmental characteristics provided by the parks still had notable inhibitory effects on visitors’ negative emotions.

### 4.4 The influence of UGS visitors’ personal characteristics on their emotions

There was significant difference in the positive emotional states of visitors with different personal characteristics during UGS visits, including age and visit duration and frequency, but not gender. However, only visitors of different age groups exhibited significant differences in their negative emotions during visit. These findings are consistent with those of White et al. (69), who found that different age groups and park visit durations yielded varying degrees of health benefits. However, the findings obtained herein did not support the findings of Ode Sang et al. (48) and Mouly et al. (63), both of which found that women had stronger associations with green spaces than men in terms of health benefits. This may be due to the different national backgrounds of studies. Reysen et al. (116) have indicated that different national backgrounds result in different emotional differences among different populations. However, the study did not focus on UGS visitors, and therefore further investigation is needed to confirm.

Although the results of one-way ANOVA indicated significant differences in emotions among individuals with different characteristics (age, frequency, and duration of visit). However, after incorporating these personal characteristics as covariates into the structural equation model for analysis, it was found that these personal characteristics did not significantly affect visitor emotions. The visitor emotions were mainly influenced by physical and psychological factors. These results are consistent with Marselle et al. (34) finding that walking time in natural environments did not significantly affect visitor emotions, but PR could significantly affect emotions. The reason for these results may be that psychological and physical factors have a much greater impact on visitor emotions than individual characteristics. In contrast, the influence of personal characteristics on emotions appears insignificant. This indicated that visitors of different age groups, visit durations, and frequencies can indeed gain varying degrees of emotional benefits in UGS. However, when they are influenced by both physical and psychological factors, a more comfortable, restorative, and preferred UGS environment significantly has a greater impact on emotions. In addition, our study did not include comprehensive personal characteristics and lacked exploration of more characteristics such as health status, levels of physical activity, and past experiences (71–73). The benefits that visitors receive from UGS may be related to these personal characteristics, which will limit the research results. Nevertheless, according to the phenomenon that there were significant differences in emotions among individuals with different characteristics (age, visit frequency, and visit duration), we still recommend that designers need to build UGS based on visitors’ characteristics.

## 5 Conclusion

This study determined that the environmental psychological and physical factors of UGS had significant impacts on the emotional well-being of visitors during the summer. A variety of subjective physical factors could affect visitor emotions by enhancing their satisfaction evaluations. However, the influences of different factors on visitor emotions varied, as did their impacts on positive and negative emotions. Positive emotions appeared to be more affected than negative emotions. Restorative perception and thermal sensation had stable and relatively substantial effects on positive and negative emotions. Air quality and sound quality was more effective in increasing positive emotions than reducing negative emotions. Moreover, environmental preference features significantly influenced only positive emotions, but could still indirectly impact negative emotions to some extent, albeit to a lesser degree. We therefore encourage relevant professionals to consider a broad range of factors, including air quality, sound, thermal, and restorative environments, as well as environmental preferences, comparing the degrees to which these factors affect visitor emotions. We have proposed specific measures and suggestions: (1) Keep the high levels of greenery, biodiversity, and naturalness, and consider appropriately combining water bodies and colorful plants; (2) Choose various types of tree species with a high crown and a large crown area and use high albedo materials to bult the road and plaza; (3) Plant tree species with dense tree crowns and dust reduction effects near noise and air pollution source in the park.

Moreover, the study had several limitations. We only investigated physical factors during the summer, and it is unclear whether similar effects are present during other seasons. The summer climate background of this study may amplify the impact of physical factors on emotions. Although there are limitations, these datasets can serve as a basis for future studies, including seasonal variations. The study here is a starting point for analysis with multiple components. Moreover, we used limited indicators, and subsequent studies should discuss more factors, such as the type of sound, SO_2_, naturalness, biodiversity, and other individual characteristics (including health status, levels of physical activity, past experiences and so on) (34, 42, 60, 71–73). We have proposed a series of recommendations to keep high levels of greenery, naturalness, and biodiversity, but some of them are based on previous studies. Therefore, we still need to further validate these conclusions to obtain more accurate and objective results.

## Data availability statement

The raw data supporting the conclusions of this article will be made available by the authors, without undue reservation.

## Ethics statement

Ethical review and approval was not required for the study of human participants in accordance with the local legislation and institutional requirements.

## Author contributions

YW: Conceptualization, Data curation, Formal Analysis, Funding acquisition, Investigation, Methodology, Resources, Visualization, Writing – original draft. JL: Conceptualization, Formal Analysis, Funding acquisition, Methodology, Resources, Writing – review & editing. JQ: Conceptualization, Formal Analysis, Methodology, Writing – original draft, Writing – review & editing. HC: Data curation, Formal Analysis, Investigation, Methodology, Writing – review & editing. KY: Conceptualization, Formal Analysis, Methodology, Supervision, Writing – review & editing. RK: Conceptualization, Funding acquisition, Methodology, Writing – review & editing.
